# KIF18B is a Prognostic Biomarker and Correlates with Immune Infiltrates in Pan-Cancer

**DOI:** 10.3389/fmolb.2021.559800

**Published:** 2021-05-24

**Authors:** Meng-jun Qiu, Qiu-shuang Wang, Qiu-ting Li, Li-sheng Zhu, Ya-nan Li, Sheng-li Yang, Zhi-fan Xiong

**Affiliations:** ^1^ Division of Gastroenterology, Liyuan Hospital, Tongji Medical College, Huazhong University of Science and Technology, Wuhan, China; ^2^ Cancer Center, Union Hospital, Tongji Medical College, Huazhong University of Science and Technology, Wuhan, China

**Keywords:** KIF18B, tumor immunity, prognosis, bioinformatics, immunecheckpoint

## Abstract

**Background:** Cancer is one of the deadliest diseases at present. Although effective screening and treatment can save lives to a certain extent, our knowledge of the disease is far from sufficient. KIF18B is a member of the kinesin-8 superfamily and plays a conserved regulatory role in the cell cycle. KIF18B reportedly functions as an oncogene in some human cancers, but the correlations between KIF18B and prognosis and immune-infiltrates in different cancers remain unclear.

**Methods:** Data were collected from the TCGA, GTEx, CCLE, TIMER, and GSEA databases. The expression difference, survival, pathological stage, DNA methylation, tumor mutation burden (TMB), microsatellite instability (MSI), mismatch repairs (MMRs), tumor microenvironment (TME), immune cell infiltration, and gene co-expression of KIF18B were analyzed using the R language software.

**Results:** KIF18B was widely upregulated in cancers, compared with normal tissues, and high KIF18B expression was associated with unfavorable prognoses. TMB, MSI, MMRs, and DNA methylation correlated with KIF18B dysregulation in cancers. KIF18B correlated closely with tumor immunity and interacted with different immune cells and genes in different cancer types.

**Conclusion:** KIF18B could be used as a prognostic biomarker for determining prognosis and immune infiltration in pan-cancer.

## 1 Introduction

Tumors have gradually become the leading cause of death in the world (Bray et al., 2018). With the continuous and relentless development of medical technology, tumor screening and treatment are constantly and significantly improving. However, due to the concealment and complexity of the occurrence and development of cancer, almost no malignant tumors can be completely cured. Although drugs targeting specific genes are considerably efficient in certain tumors, no satisfactory outcome has been reached yet. In recent years, the roles of the tumor microenvironment (TME) and host immunity have become increasingly important, and the emergence of immune checkpoint inhibitors (ICIs) has brought promising perspectives to more patients (Topalian et al., 2016). With this increase in clinical applications, more effective targets must be found.

Kinesin superfamily proteins (KIFs) are a group of proteins with highly conserved motor domains that can polymerize and dissociate from microtubules and use the energy released by ATP hydrolysis to promote the directional movement of KIFs (Miki et al., 2005). The motor ability of KIFs plays a vital role in mitosis, vesicle trafficking, and the maintenance of cell polarity, among others (Hirokawa et al., 1998; Sharp et al., 2000; Hirokawa and Takemura, 2005)*.* KIF18B, a member of the kinesin-8 proteins, is involved in the separation of chromosomes and spindle localization during mitosis (Du et al., 2010; McHugh et al., 2018). In recent years, studies have shown that KIF18B participates in the growth and development of a variety of cancers (Wu et al., 2018; Zhong et al., 2019; Li et al., 2020; Yang et al., 2020). However, to date, the expression pattern, prognostic significance, and biological function of KIF18B in cancer have not been elucidated fully.

In this study, we conducted a comprehensive analysis of KIF18B at the transcription level, tumor mutation burden (TMB) level, DNA methylation level, microsatellite instability (MSI) level, mismatch repairs (MMRs) level, TME level, clinical prognosis level, and more.

## 2 Materials and Methods

### 2.1 The Cancer Genome Atlas (TCGA) Database

TCGA is a large-scale sequencing-based genomic analysis technology for the understanding of the molecular mechanisms of cancer (https://cancergenome.nih.gov) (AuthorAnonymous, 2018). We downloaded the expression profile data, TMB data, MSI data, and clinical data of 33 tumors from TCGA.

### 2.2 The Genotype-Tissue Expression (GTEx) Program

The GTEx program has collected and studied more than 7,000 autopsy samples from 449 human donors who were healthy during their lifetime, covering 44 organizations (http://commonfund.nih.gov/GTEx/) (Battle et al., 2017). GTEx observed gene expression patterns of almost all transcribed genes. Given that there are fewer normal samples in TCGA, we integrated normal tissue data from GTEx with tumor tissue data from TCGA to analyze the expression differences of KIF18B.

### 2.3 The Cancer Cell Line Encyclopedia (CCLE) Database

We used CCLE, an online database that provides an analysis and visualization of more than 1,100 cell lines (https://portals.broadinstitute.org/ccle) (Ghandi et al., 2019), to assess the expression level of KIF18B in the tumor cell lines of 21 tissues.

### 2.4 Tumor IMmune Estimation Resource (TIMER) Database

TIMER, a web server used for the comprehensive analysis of tumor-infiltrating immune cells (https://cistrome.shinyapps.io/timer/) (Li et al., 2017), was used to analyze the correlation of KIF18B expression with the abundance of immune infiltrates.

### 2.5 Gene Set Enrichment Analysis (GSEA) Database

GSEA is based on the existing knowledge of gene location, function, and biological significance and is used to build a database containing multiple functional gene sets (https://www.gsea-msigdb.org/gsea/index.jsp) (Subramanian et al., 2005). Samples were separated into a high group and a low group according to the median expression of KIF18B, and the GSEA software was used to enrich the gene ontology (GO) and Kyoto encyclopedia of genes and genomes (KEGG) pathway.

### 2.6 Statistical Analysis

The Perl script was used to organize and normalize data matrixes, classifying them into cancerous tissues and adjacent tissues and extracting the expression of KIF18B. The analyses of the expression difference, survival, pathological stage, DNA methylation, TMB, MSI, MMRs, TME, immune cell infiltration, and gene co-expression of KIF18B were performed using the R language (Version 3.6.3) (https://www.r-project.org/) for statistical analysis, including the “ggpubr”, “limma”, “survival”, “survminer”, “fmsb”, “ggExtra”, “ggplot2”, “e1071”, “org.Hs.eg.db”, “clusterProfiler”, “enrichplot”, and “forestplot” projects.

## 3 Results

### 3.1 Upregulation of KIF18B mRNA in Cancers

**FIGURE 1:**
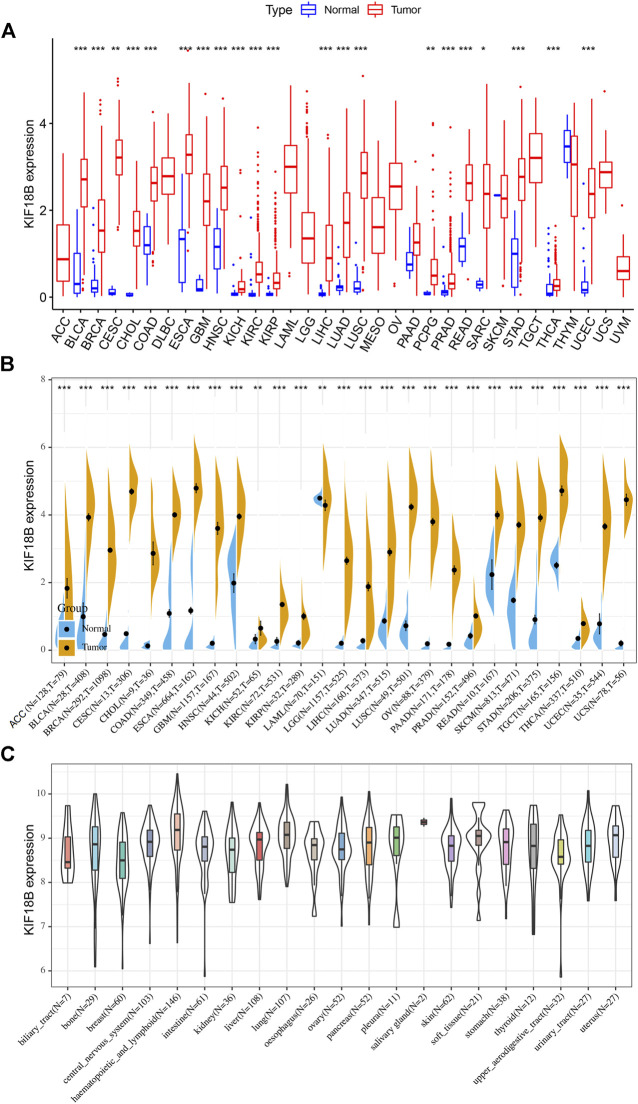
The transcription levels of KIF18B in various cancers. **(A)** The evaluation of KIF18B mRNA in 33 types of cancers from TCGA database. **(B)** The evaluation of KIF18B mRNA in 27 types of cancers by integrating normal tissue data from GTEx with tumor tissue data from TCGA. **(C)** The evaluation of KIF18B mRNA in the cancer cell lines of 21 tissues using the CCLE database. * indicates *p* < 0.05, ** indicates *p* < 0.01, *** indicates *p* < 0.001.

We extracted the mRNA levels of KIF18B from 33 cancer types in TCGA and plotted a box diagram of the expression of KIF18B in cancerous and adjacent tissues. As shown in Figure 1A, the expression of KIF18B mRNA in most cancers was different from that in normal tissues. KIF18B mRNA levels increased significantly in bladder urothelial carcinoma (BLCA), breast invasive carcinoma (BRCA), cervical squamous cell carcinoma and endocervical adenocarcinoma (CESC), cholangiocarcinoma (CHOL), colon adenocarcinoma (COAD), esophageal carcinoma (ESCA), glioblastoma multiforme (GBM), head and neck squamous cell carcinoma (HNSC), kidney chromophobe (KICH), kidney renal clear cell carcinoma (KIRC), kidney renal papillary cell carcinoma (KIRP), liver hepatocellular carcinoma (LIHC), lung adenocarcinoma (LUAD), lung squamous cell carcinoma (LUSC), pheochromocytoma and paraganglioma (PCPG), prostate adenocarcinoma (PRAD), rectum adenocarcinoma (READ), sarcoma (SARC), stomach adenocarcinoma (STAD), thyroid carcinoma (THCA), and uterine corpus endometrial carcinoma (UCEC) and increased inconsiderably in pancreatic adenocarcinoma (PAAD). But decrease did not change substantially in thymoma (THYM) and skin cutaneous melanoma (SKCM). Because the transcription levels of the corresponding adjacent tissues in adrenocortical carcinoma (ACC), lymphoid neoplasm diffuse large B-cell lymphoma (DLBC), acute myeloid leukemia (LAML), brain lower grade glioma (LGG), mesothelioma (MESO), ovarian serous cystadenocarcinoma (OV), testicular germ cell tumors (TGCT), uterine carcinosarcoma (UCS), and uveal melanoma (UVM) were unavailable, we integrated normal tissue data into GTEx data. As shown in Figure 1B, KIF18B mRNA levels were also higher in ACC, LGG, OV, TGCT, and UCS. Additionally, KIF18B was generally higher in the tumor cell lines of 21 tissues (Figure 1C).

### 3.2 High KIF18B Expression was Associated with Unfavorable Outcomes in Cancers

**FIGURE 2:**
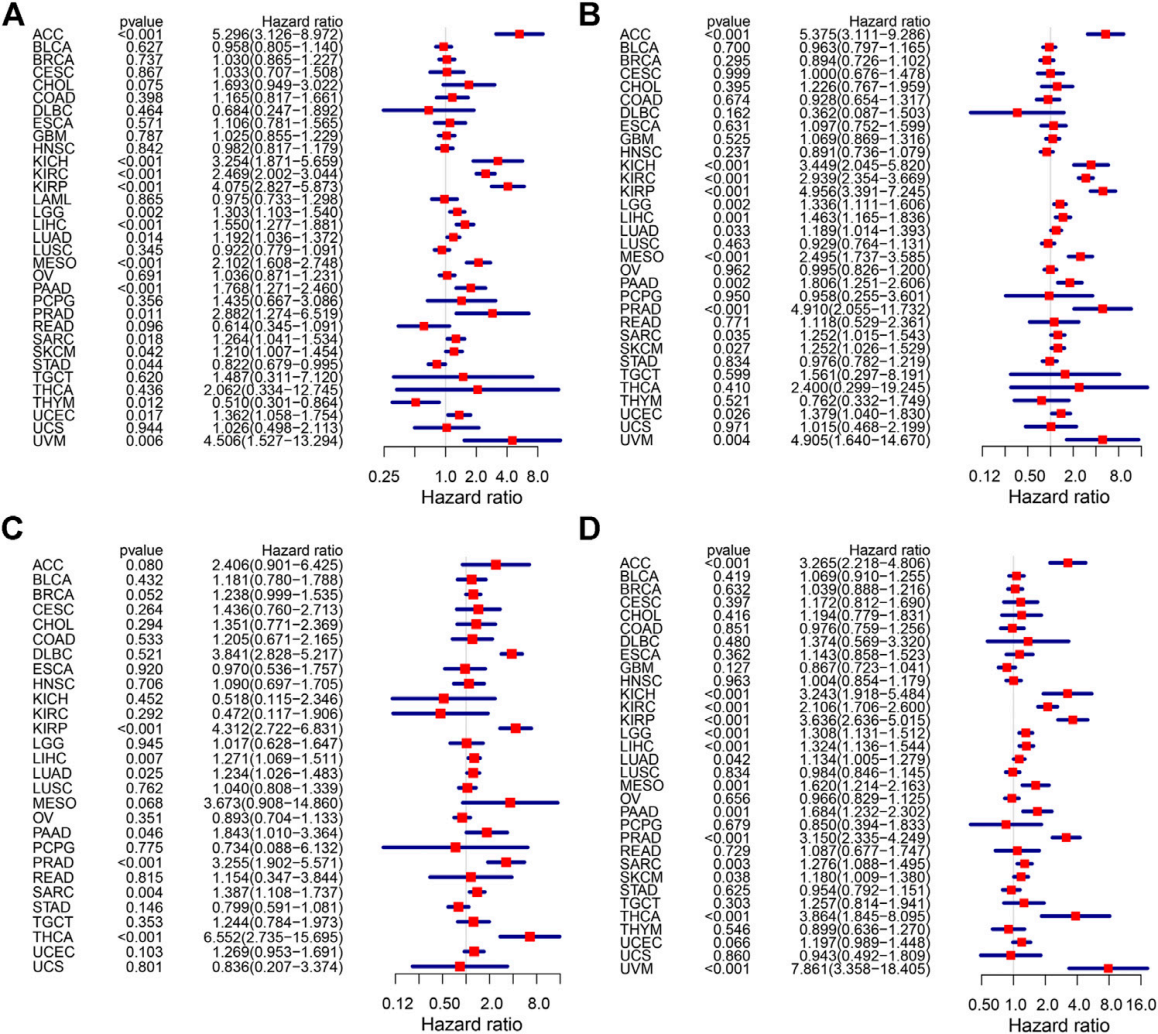
The effect of KIF18B on the prognosis of various cancers using Cox proportional hazards models. **(A)** The effect of KIF18B on OS in 33 types of cancers. **(B)** The effect of KIF18B on DSS in 33 types of cancers. **(C)** The effect of KIF18B on DFS in 33 types of cancers. **(D)** The effect of KIF18B on PFI in 33 types of cancers.

We integrated the KIF18B mRNA level with the overall survival (OS), disease-specific survival (DSS), disease-free survival (DFI), and progression-free interval (PFI) of the 33 cancer types and used the Kaplan-Meier (KM) survival curve and Cox proportional hazards models to evaluate the effect of KIF18B on cancer prognosis. Cox regression analyses were displayed in forest charts. The correlations of KIF18B with OS, DSS, DFI, and PFI in different cancers were visually observed. Highly expressed KIF18B correlated negatively with OS in ACC (*p* < 0.001), KICH (*p* < 0.001), KIRC (*p* < 0.001), KIRP (*p* < 0.001), LGG (*p* = 0.002), LIHC (*p* < 0.001), LUAD (*p* = 0.014), MESO (*p* < 0.001), PAAD (*p* < 0.001), PRAD (*p* = 0.011), SARC (*p* = 0.018), SKCM (*p* = 0.042), STAD (*p* = 0.044), UCEC (*p* = 0.017), and UVM (*p* = 0.006) and positively with OS in THYM (*p* = 0.012) and STAD (*p* = 0.044) (Figure 2A), negatively with DSS in ACC (*p* < 0.001), KICH (*p* < 0.001), KIRC (*p* < 0.001), KIRP (*p* < 0.001), LGG (*p* = 0.002), LIHC (*p* = 0.001), LUAD (*p* = 0.033), MESO (*p* < 0.001), PAAD (*p* = 0.002), PRAD (*p* < 0.001), SARC (*p* = 0.035), SKCM (*p* = 0.027), UCEC (*p* = 0.026), and UVM (*p* = 0.004) (Figure 2B), negatively with DFI in KIRP (*p* < 0.001), LIHC (*p* = 0.007), LUAD (*p* = 0.025), PAAD (*p* = 0.046), PRAD (*p* < 0.001), SARC (*p* = 0.004), and THCA (*p* < 0.001) (Figure 2C), and negatively with PFI in ACC (*p* < 0.001), KICH (*p* < 0.001), KIRC (*p* < 0.001), KIRP (*p* < 0.001), LGG (*p* < 0.001), LIHC (*p* < 0.001), LUAD (*p* = 0.042), MESO (*p* = 0.001), PAAD (*p* = 0.001), PRAD (*p* < 0.001), SARC (*p* = 0.003), SKCM (*p* = 0.038), THCA (*p* < 0.001), and UVM (*p* < 0.001) (Figure 2D).

**FIGURE 3:**
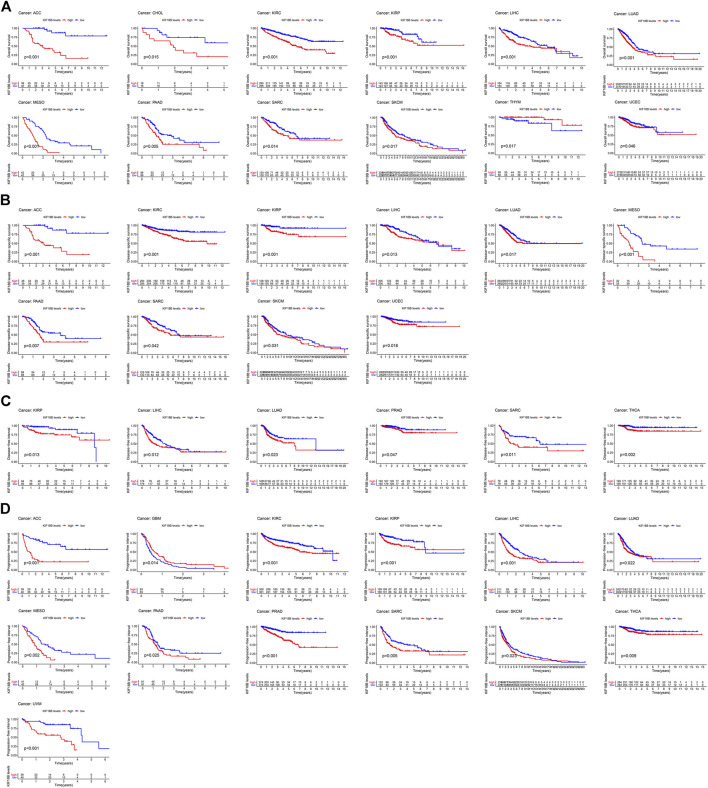
The survival curve of KIF18B in various cancers using Kaplan-Meier methods and the log-rank test. Values of *p* < 0.05 were considered and displayed. **(A)** The survival curve of KIF18B for OS in 12 types of cancers. **(B)** The survival curve of KIF18B for DSS in 10 types of cancers. **(C)** The survival curve of KIF18B for DFS in 6 types of cancers. **(D)** The survival curve of KIF18B for PFI in 13 types of cancers.

For the visualization of the impact of KIF18B on prognosis using the KM curve, and the filter condition was *p* < 0.05. As shown in Figure 3A, higher levels of KIF18B mRNA indicated worse OS in ACC (*p* < 0.001), CHOL (*p* = 0.015), KIRC (*p* < 0.001), KIRP (*p* = 0.001), LIHC (*p* = 0.001), LUAD (*p* = 0.001), MESO (*p* < 0.001), PAAD (*p* = 0.005), SARC (*p* = 0.014), SKCM (*p* = 0.017), and UCEC (*p* = 0.046), while the opposite result was observed in THYM (*p* = 0.017).

Higher levels of KIF18B mRNA were also linked with worse DSS in ACC (*p* < 0.001), KIRC (*p* < 0.001), KIRP (*p* < 0.001), LIHC (*p* = 0.013), LUAD (*p* = 0.017), MESO (*p* < 0.001), PAAD (*p* = 0.007), SARC (*p* = 0.042), SKCM (*p* = 0.031), and UCEC (*p* = 0.018) (Figure 3B), worse DFI in KIRP (*p* = 0.013), LIHC (*p* = 0.012), LUAD (*p* = 0.023), PRAD (*p* = 0.047), SARC (*p* = 0.011), and THCA (*p* = 0.002) (Figure 3C), and worse PFI in ACC (*p* < 0.001), KIRC (*p* < 0.001), KIRP (*p* < 0.001), LIHC (*p* < 0.001), LUAD (*p* = 0.022), MESO (*p* = 0.002), PAAD (*p* = 0.025), PRAD (*p* < 0.001), SARC (*p* = 0.005), SKCM (*p* = 0.023), THCA (*p* = 0.009), and UVM (*p* < 0.001), while better PFI in GBM (*p* = 0.014) (Figure 3D).

### 3.3 Correlations of KIF18B Expression with Pathological Stages in Cancers

**FIGURE 4:**
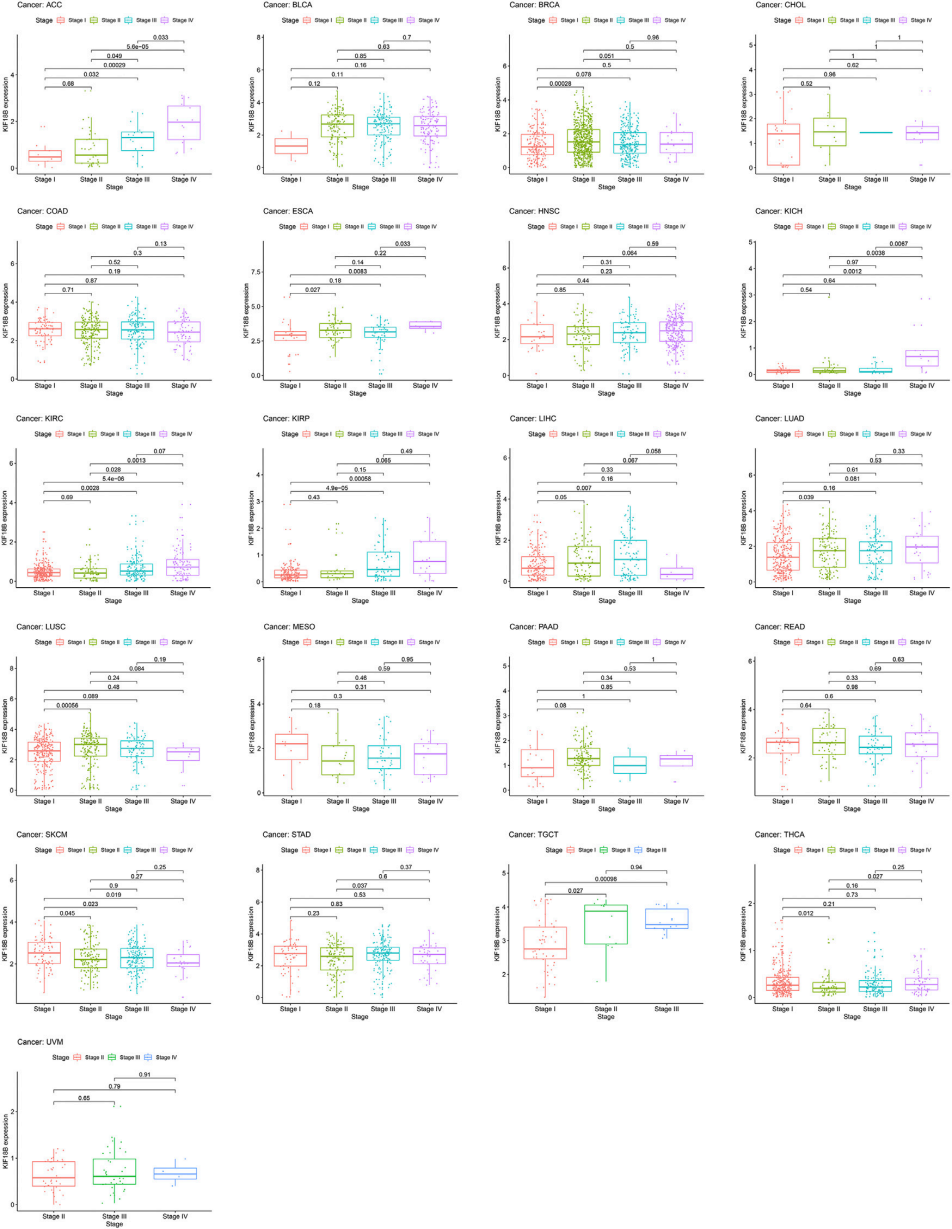
The correlations between KIF18B expression and pathological stages in various cancers.

To assess whether KIF18B mRNA was expressed at different levels at various cancer stages, we extracted pathological stages, including stages I, II, III, and IV, from 33 tumors and integrated them with KIF18B mRNA levels. Figure 4 shows that there were differences in KIF18B expression between stages I and II in BRCA, ESCA, LUAD, LUSC, SKCM, TGCT, and THCA, between stages I and III in ACC, KIRC, KIRP, LIHC, SKCM, and TGCT, between stages I and IV in ACC, ESCA, KICH, KIRC, KIRP, and SKCM, between stages II and III in ACC, KIRC, and STAD, between stages II and IV in ACC, KICH, KIRC, and THCA, and between stages III and IV in ACC, ESCA, and KICH. However, there was no difference in KIF18B expression between different pathological stages in BLCA, CHOL, COAD, HNSC, MESO, PAAD, READ, and UVM. In addition, no pathological staging records were detected in CESC, DLBC, GBM, LAML, LGG, OV, PCPG, PRAD, SARC, THYM, UCEC, and UCS.

### 3.4 Correlations of KIF18B Expression with TMB and MSI

TMB refers to the number of somatic mutations that occur on the average 1 Mb base in the coding region of the tumor cell genome (Campbell et al., 2017). MSI represents the random changes in the length of microsatellites in tumor cells due to the insertion or deletion of repeating units, compared to normal cells, and the emergence of new microsatellite alleles (Cortes-Ciriano et al., 2017). We calculated the TMB and MSI of each sample in all tumors and then analyzed the relationship between KIF18B expression and TMB and MSI in different tumors using the Spearman rank correlation coefficient and displayed it as a radar chart.

**FIGURE 5:**
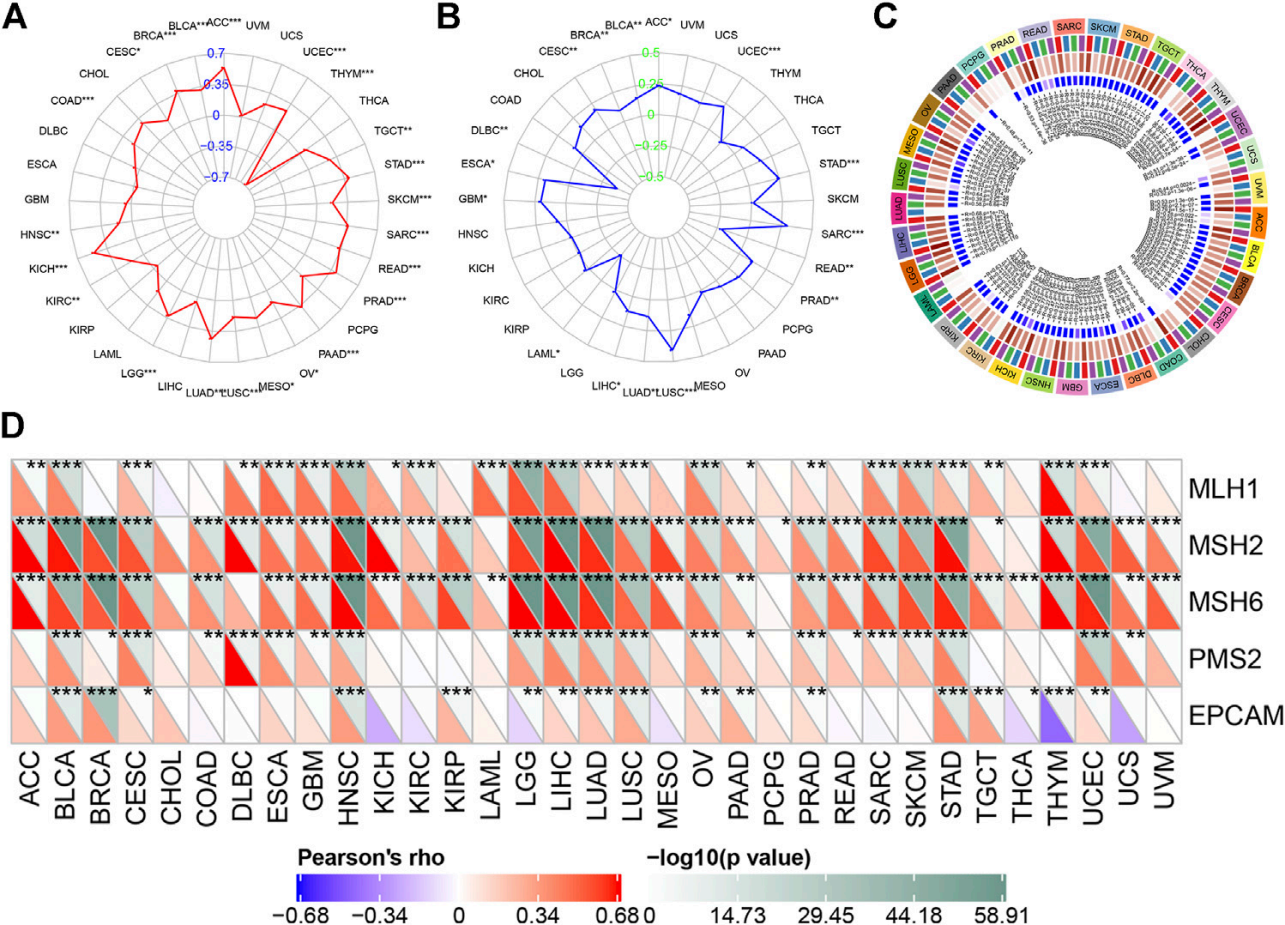
The correlations between KIF18B expression and tumor mutation burden (TMB), microsatellite instability (MSI), DNA methylation, and mismatch repairs (MMRs) in various cancers. **(A)** The correlations between KIF18B expression and TMB in 33 types of cancers. **(B)** The correlation between KIF18B and MSI in 33 types of cancers. **(C)** The correlations between KIF18B and four methyltransferases (DNMT1: red, DNMT2: blue, DNMT3A: green, and DNMT3B: purple) in 33 types of cancers. **(D)** The correlation between KIF18B and five MMRs related genes in 33 types of cancers.

As shown in Figure 5A, the high expressions of KIF18B correlated significantly positively with TMB in ACC, BLCA, BRCA, CESC, COAD, HNSC, KICH, KIRC, LGG, LUAD, LUSC, MESO, OV, PAAD, PRAD, READ, SARC, SKCM, STAD, TGCT, and UCEC but markedly negatively in THYM (Supplementary Table S1). Figure 5B shows that the high expressions of KIF18B correlated significantly positively with MSI in ACC, BLCA, BRCA, CESC, ESCA, GBM, LIHC, LUAD, LUSC, PRAD, SARC, STAD, and UCEC but considerably negatively in DLBC, LAML, and READ (Supplementary Table S2).

### 3.5 Correlations of KIF18B Expression with DNA Methylation and MMRs

DNA methylation is a form of chemical modification of DNA that can change genetic performance without changing the DNA sequence (Perri et al., 2017). We evaluated the relationship between four methyltransferases, including DNMT1: red, DNMT2: blue, DNMT3A: green, and DNMT3B: purple, and KIF18B expression. As shown in Figure 5C, KIF18B expressions associated positively with all four methyltransferases in 20 types of cancer but did not correlate with any of the four methyltransferases in PCPG or with DNMT2 in PRAD, PAAD, LUAD, LAML, KIRC, COAD, CHOL, UVM, UCS, UCEC, and THCA, DNMT3A in PAAD, LAML, DLBC, CHOL, and UCS, or DNMT3B in LAML and CHOL. MMRs are intracellular mismatch repair mechanisms. The loss of key gene functions in these mechanisms can lead to DNA replication errors that cannot be repaired, eventually causing higher somatic mutations (Kunkel and Erie, 2005). We evaluated the relationship between five MMRs-related genes, including MLH1, MSH2, MSH6, PMS2, and EPCAM, and KIF18B expression. As shown in Figure 5D, KIF18B expressions correlated significantly positively with MLH1 in 23 types of cancer, with MSH2 in 30 types of cancer, with MSH6 in 30 types of cancer, with PMS2 in 21 types of cancer, and with EPCAM in 14 types of cancer. Additionally, KIF18B expressions correlated significantly negatively with EPCAM in LGG, THCA, and THYM.

### 3.6 Correlations of KIF18B Expression with TME

**FIGURE 6:**
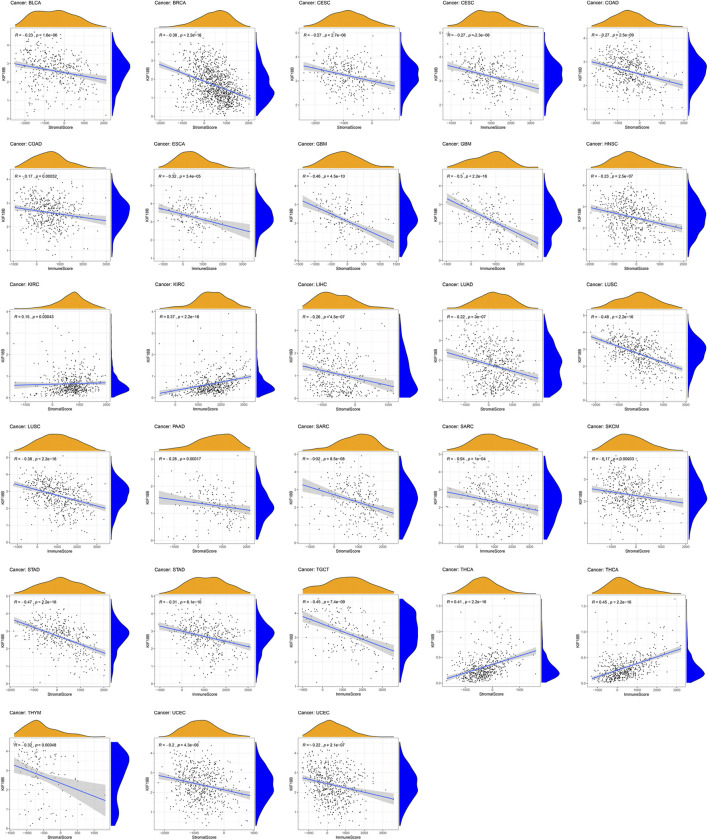
The correlations between KIF18B expression and the stromal and immune scores of tumor microenvironments in various cancers. Values of *p* < 0.001 were considered and displayed.

TME is the local biological environment where tumors are located. Abnormal alterations to this environment can induce changes, such as promoting tumor cell proliferation, invasion, metastasis, and inhibiting apoptosis, to the biological behavior of tumor cells (Pitt et al., 2016; Locy et al., 2018). We obtained the stromal and immune scores of each sample in all tumors and assessed the relationship between KIF18B expression and both scores in TME using the Spearman rank correlation coefficient (Supplementary Table S3). We visualized the results at *p* < 0.001. As shown in Figure 6, the high expressions of KIF18B correlated significantly negatively with stromal cells in BLCA, BRCA, CESC, COAD, GBM, HNSC, LIHC, LUAD, LUSC, PAAD, SARC, SKCM, STAD, THYM, and UCEC, and immune cells in CESC, COAD, ESCA, GBM, LUSC, SARC, STAD, TGCT, and UCEC but the correlations between high KIF18B levels and stromal and immune scores in KIRC and THCA were positive.

### 3.7 Correlations of KIF18B Expression with Immune Cell Infiltration

We obtained the content of 22 specific immune cells in each sample of all tumors and observed the relationship between KIF18B expression and these contents using the Spearman rank correlation coefficient (Supplementary Table S4). We visualized the results at *p* < 0.001. As shown in Supplementary Figure S1, the high expressions of KIF18B correlated significantly negatively with T cells CD4 memory resting in UCEC (R = −0.18), TGCT (R = −0.27), LUSC (R = −0.22), LUAD (R = −0.29), KIRC (R = −0.19), GBM (R = −0.3), COAD (R = −0.18), and BRCA (R = −0.19), but significantly positively with T cells CD4 memory activated in UCEC (R = 0.26), THCA (R = 0.37), STAD (R = 0.21), LUAD (R = 0.36), LIHC (R = 0.21), KIRP (R = 0.23), KIRC (R = 0.2), COAD (R = 0.19), and BRCA (R = 0.26). Highly expressed KIF18B also correlated significantly positively with T cells follicular helper in UCEC (R = 0.25), THCA (R = 0.34), STAD (R = 0.35), OV (R = 0.2), LUSC (R = 0.2), LUAD (R = 0.2), LIHC (R = 0.37), KIRP (R = 0.25), KIRC (R = 0.21), HNSC (R = 0.15), BRCA (R = 0.18), and BLCA (R = 0.22) and T cells regulatory (Tregs) in PRAD and KIRC but significantly negatively with Tregs in UCEC (R = −0.21), SKCM (R = −0.19), DLBC (R = −0.51), and BLCA (R = −0.26). Furthermore, the high expressions of KIF18B correlated significantly negatively with macrophages M0 in THYM (R = −0.55) and KIRP (R = −0.23) but substantially positively in STAD (R = 0.35), SARC (R = 0.26), PAAD (R = 0.28), LUSC (R = 0.18), LUAD (R = 0.4), LIHC (R = 0.22), BRCA (R = 0.3), and ACC (R = 0.6), significantly negatively with macrophages M1 in THYM (R = −0.44) but markedly positively in UCEC (R = 0.32), THCA (R = 0.34), STAD (R = 0.22), LUSC (R = 0.2), LUAD (R = 0.42), KIRP (R = 0.25), KIRC (R = 0.17), BRCA (R = 0.26), and BLCA (R = 0.2), significantly negatively with macrophages M2 in THYM (R = −0.63), THCA (R = −0.32), STAD (R = −0.17), LIHC (R = −0.33), KIRP (R = −0.3), and KIRC (R = −0.21), but considerably positively in TGCT (R = 0.45) and GBM (R = 0.33), significantly negatively with dendritic cells resting in STAD (R = −0.24), LUSC (R = −0.23), LUAD (R = −0.37), HNSC (R = −0.31), COAD (R = −0.2), and BRCA (R = −0.13), but remarkably positively in THYM (R = 0.65) and LIHC (R = 0.19), and significantly negatively with mast cells resting in THYM (R = −0.56), STAD (R = −0.3), LUAD (R = −0.43), KIRC (R = −0.27), HNSC (R = −0.16), BRCA (R = −0.34), BLCA (R = −0.23), and ACC (R = −0.5).

### 3.8 Correlations of KIF18B Expression with Immune-Related Factors

**FIGURE 7:**
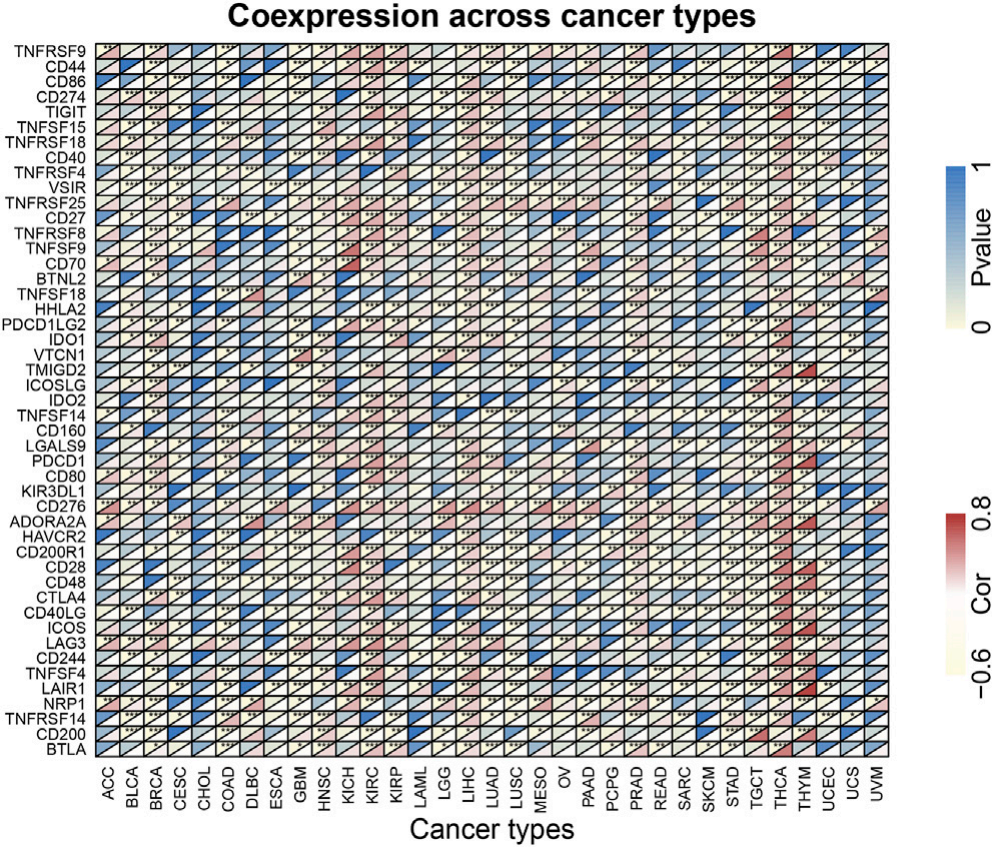
The correlations between KIF18B expression and immune-related factors in various cancers. * indicates *p* < 0.05, ** indicates *p* < 0.01, *** indicates *p* < 0.001.

To further explore whether KIF18B could be used as a potential target for immune checkpoints in cancer, we examined the relationship between KIF18B expression and some known immune-related factors, displaying the outcome as a heat map. As shown in Figure 7, KIF18B correlated negatively with 9 immune-related factors and positively with 27 immune-related factors in BRCA, negatively with 21 immune-related factors and positively with 9 immune-related factors in COAD, negatively with 24 immune-related factors and positively with 6 immune-related factors in GBM, positively with 29 immune-related factors and negatively with 1 immune-related factor in HNSC, positively with 33 immune-related factors and negatively with 4 immune-related factors in KIRC, positively with 39 immune-related factors and negatively with 1 immune-related factor in LIHC, positively with 31 immune-related factors and negatively with 5 immune-related factors in PRAD, positively with 8 immune-related factors and negatively with 31 immune-related factors in TGCT, positively with 42 immune-related factors in THCA, and positively with 14 immune-related factors and negatively with 21 immune-related factors in THCA.

### 3.9 Predicted Functions of KIF18B in Cancers

**FIGURE 8:**
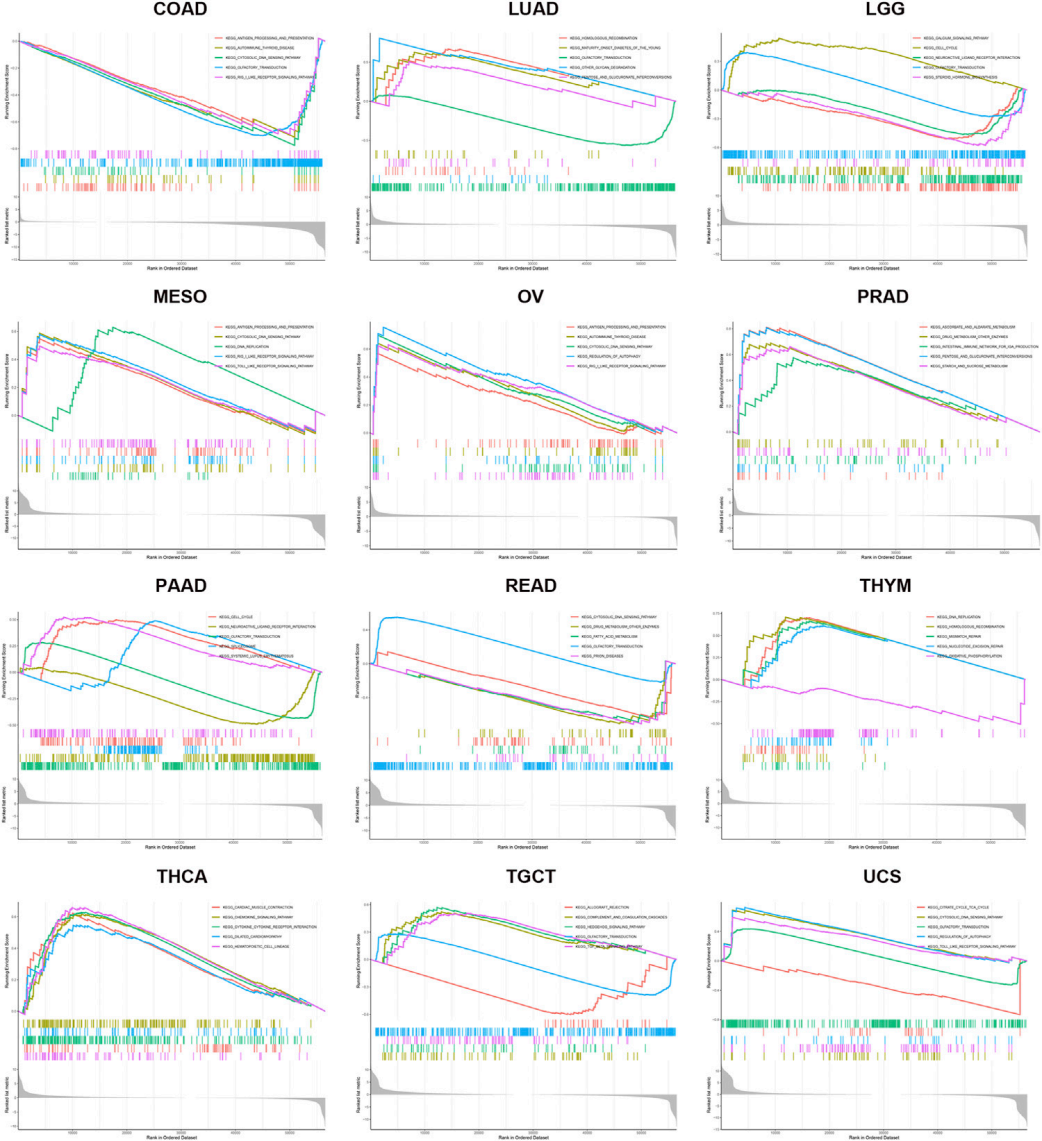
KEGG enrichment analysis of KIF18B in various cancers. Values of *p* < 0.05 and results higher than 5 were considered and displayed.

GSEA was used for the GO and KEGG analyses of KIF18B positively co-expressed genes in pan-cancers. GO enrichment analysis predicted the functional roles of target host genes based on three aspects: biological processes, cellular components, and molecular functions. Results at *p* < 0.05 were screened out, and the enrichment results higher than five were visualized. GO analysis suggested that these genes were mainly concentrated in the chromosome, spindle, and microtubule and that they were primarily involved in cell cycle-related activities and regulation (Supplementary Figure S2; Supplementary Tables S5–S37). Per the KEGG analysis, KIF18B in pan-cancer tissues participated largely in antigen processing and presentation, cell cycle, cytosolic DNA sensing pathway, regulation of autophagy, and toll-like receptor signaling pathway (Figure 8; Supplementary Tables S38–S70).

## 4 Discussion

Kinesin is a motor molecule that exists in all eukaryotes. Most newly synthesized proteins in cells move through molecular motors along directional cytoskeletal fibers to their appropriate destinations. Substances are precisely transported and unloaded to the corresponding parts to perform specific functions, which is essential to maintain the morphology and function of cells, especially during cell division (Mazumdar and Misteli, 2005; Wu et al., 2006). Kinesin is precise because it plays a vital role in the regulation of cytokinesis, spindle formation, and chromosome assembly and arrangement; so, its abnormal expression can easily cause abnormal cell replication and differentiation, which can lead to cancer. A variety of KIFs are abnormally expressed in various tumor cells, and their role in promoting cancer has also been studied to varying degrees (Wang et al., 2017; Hu et al., 2019; Jungwirth et al., 2019; Li et al., 2019; Xu et al., 2019). Since tumors are gene-related diseases with abnormal cell proliferation, targeting kinesin therapy could be a new strategy for tumor treatment. KIF18B is located on chromosome 17q21.31 and consists of 17 exons. The protein is a novel dynamics regulatory protein that regulates astral microtubule length and life in space conformation (Walczak et al., 2016). The underlying molecular mechanisms of KIF18B dysregulation in tumors have not been elucidated fully. Considering the potential prognostic value and biological function of KIF18B, it is essential to explore the fundamental mechanism of its dysregulation in pan-cancer.

In this study, we first revealed that KIF18B was upregulated at mRNA levels in ACC, BLCA, BRCA, CESC, CHOL, COAD, ESCA, GBM, HNSC, KICH, KIRC, KIRP, LIHC, LUAD, LUSC, LGG, PCPG, PRAD, READ, SARC, STAD, THCA, UCEC, OV, TGCT, and UCS cancer tissues, compared with corresponding normal tissues, suggesting that KIF18B might act as an oncogene in pan-cancers. Based on survival curves, including OS, DFI, DSS, and PFI, KIF18B mRNA expression demonstrated reliable diagnostic value, indicating that KIF18B is a potentially promising biomarker for pan-cancer diagnosis. We further explored the clinical significance of KIF18B mRNA expression in pan-cancers and revealed that KIF18B mRNA expressions were higher in advanced pathological stages (III and IV) in ACC, KIRC, KIRP, KICH, LIHC, SKCM, TGCT, ESCA, and STAD, which pointed to KIF18B playing a role in the carcinogenesis and progression of pan-cancer.

TMB and MSI are very common in cancer and may have a profound impact on tumor phenotype and patient survival (Hause et al., 2016; Samstein et al., 2019). In the current study, the upregulation of KIF18B mRNA correlated significantly positively with TMB and MSI in ACC, BLCA, BRCA, CESC, LUAD, LUSC, PRAD, SARC, STAD, and UCEC, but negatively in THYM. Therefore, we speculate that the high neo-antigen load causing the aberrant KIF18B expression and dysregulation in pan-cancer is a positive note for biomarkers of new antigens. DNA methylation and MMRs are linked to the activation of oncogenes and the inhibition of tumor suppressor genes. In our study, KIF18B expression correlated positively with the methylation and MMRs of most cancers, suggesting that DNA methylation and MMRs may change the expression of KIF18B.

There are several immune cells in the TME, and these can be further divided into immune-killer cells linked to tumor-killing and immunosuppressive cells associated with tumor-escape immune surveillance. Tumor immune escape is an important mechanism that imparts malignant features to tumors. Based on the principle of immune escape, the development of specific tumor immunotherapy has gradually become a research hotspot in the field of cancer. Immunotherapy of tumors, including monoclonal antibody-type ICIs, therapeutic antibodies, cancer vaccines, cell therapy, small molecule inhibitors, among others (O'Donnell et al., 2019; Yang, 2015), is a treatment that restarts and maintains the tumor-immune cycle, restores the body’s anti-tumor immune response, thus, controlling and clearing tumors. The higher the stromal score and immune score, the fewer the cancer cells. Here, our results showed that KIF18B expression correlated significantly negatively with the purity of stromal cells and immune cells in CESC, COAD, GBM, LUSC, SARC, STAD, and UCEC. KIF18B expression also correlated markedly negatively with infiltrating levels of T cells CD4 memory resting in eight types of cancer, macrophages M2 in six types of cancer, dendritic cells resting in six types of cancer, and mast cells resting in eight types of cancer. Moreover, KIF18B expression correlated considerably positively with infiltrating levels of T cells CD4 memory activated in nine types of cancer, T cells follicular helper in 12 types of cancer, macrophages M0 in eight types of cancer, and macrophages M1 in nine types of cancer. The correlation between KIF18B and tumor-associated macrophages (TAMs) revealed that KIF18B possibly plays an immunosuppressive role in cancers by promoting the presentation of tumor antigens rather than secreting immune-inflammatory mediators and that KIF18B could potentially also activate Tregs and induce T cell exhaustion, pointing to a likely regulatory role of KIF18B in tumor immunology. These findings require further validation and experimentation.

To further evaluate whether KIF18B may have an immune-related role in cancer, we collected 47 common immune-related genes. Among them, CD274 (PD-L1) is the hottest immune checkpoint currently studied. As an inhibitory ligand, after binding to its receptor PD-1, PD-L1 negatively regulates T cell proliferation and cytokine secretion at a certain threshold of T cell receptor attack, impairs T cell immune function in the TME, and maintains peripheral tolerance (Parsa et al., 2007; Ansell et al., 2015). According to our findings, the PD-L1 function correlated highly with KIF18B expression in BLCA, BRCA, KIRC, LIHC, LUAD, OV, PAAD, PCPG, STAD, and THCA. Lag-3 acts as a negative regulator of T cell activation, binding major histocompatibility complex class II. Blocking Lag-3 can improve cytotoxic T lymphocyte proliferation and effector function. Lag-3 is also co-expressed with PD-1 in tumor-infiltrating lymphocytes in tumors and synergizing with PD-1 blockade may improve tumor control or regression (Grosso et al., 2007; Woo et al., 2012). In this study, Lag-3 correlated highly with KIF18B expression in ACC, BLCA, BRCA, COAD, GBM, HNSC, KICH, KIRC, KIRP, LGG, LIHC, LUAD, OV, PAAD, PRAD, READ, STAD, THCA, and UCEC. KIF18B, PD-L1, and Lag-3 showed the same expression trends in tumors, suggesting that they may share some pathways that promote tumor aggressiveness. HAVCR2 (Tim-3), a crucial surface protein on exhausted T cells and anti-Tim-3 antibodies can affect the phenotype of myeloid cells in the TME (Huang et al., 2015). Tim-3 can also suppress immune response indirectly by abrogating the acquisition of an M2-like phenotype in TAMs (Dardalhon et al., 2010; Kurtulus et al., 2019). KIF18B expression and Tim-3 are mutually exclusive in CESC, COAD, ESCA, GBM, KIRP, LAML, LUSC, PCPG, SARC, STAD, and TGCT. Therefore, targeting KIF18B may be an option for patients who are ineligible for Tim-3 immunotherapy.

In animal research, Castle et al. performed whole-exome sequencing on B16F10 murine melanoma and inoculated the identified mutant gene-encoding peptide into mice for tumor immunization. They found that the mice immunized with mutant KIF18B peptide had weaker tumor progression and higher survival rates, which provided an experimental basis for our research results (Castle et al., 2012). In short, these correlations could indicate potential mechanisms and suggest that KIF18B plays a vital role in the recruitment and regulation of the immune system in pan-cancer. Furthermore, we explored the basic biological function and molecular mechanisms that underpin the potential clinical value of KIF18B in pan-cancers. In addition to the known “cell cycle” as the most important signaling pathway for KIF18B co-expressed genes, we determined that KIF18B was involved in antigen processing and presentation, cytosolic DNA sensing pathway, regulation of autophagy, toll-like receptor signaling pathway, and many more in various cancers. These findings could help predict the possibility of the use of KIF18B as a new immune target. Of course, our results were based on bioinformatics analysis, meaning that there were no experiments to verify our claims, and transcriptomics levels do not reflect protein expression levels or activity. However, our findings provided new insights into the development and treatment of KIF18B in cancer. We intend to perform in-depth studies to validate our current conclusions in future research.

In summary, KIF18B was upregulated in pan-cancer tissues, compared with corresponding normal tissues. Additionally, the high expression of KIF18B was associated with unfavorable clinicopathological features and independently predicted poor prognosis in pan-cancer. Also, TMB, MSI, MMRs, and DNA methylation might contribute to KIF18B dysregulation in cancers, and KIF18B is closely linked to tumor immunity and may be a potential therapeutic target for immunotherapy.

## Data Availability

The datasets presented in this study can be found in online repositories. The names of the repository/repositories and accession number(s) can be found in the article/Supplementary Material.
