# Supplementary material for: KIF18B is a Prognostic Biomarker and Correlates with Immune Infiltrates in Pan-Cancer
Source: Front Mol Biosci. 2021 May 24;8:559800. doi: 10.3389/fmolb.2021.559800 (PMC8182049; doi:10.3389/fmolb.2021.559800)

**Supplementary Materials for Figures**

**KIF18B is a prognostic biomarker and correlates with immune infiltrates in** **pan-cancer**

**Supplementary Figure 1.** The correlation between KIF18B expression and immune cell infiltration in various cancers. Values of *P* < 0.001 were considered and displayed.


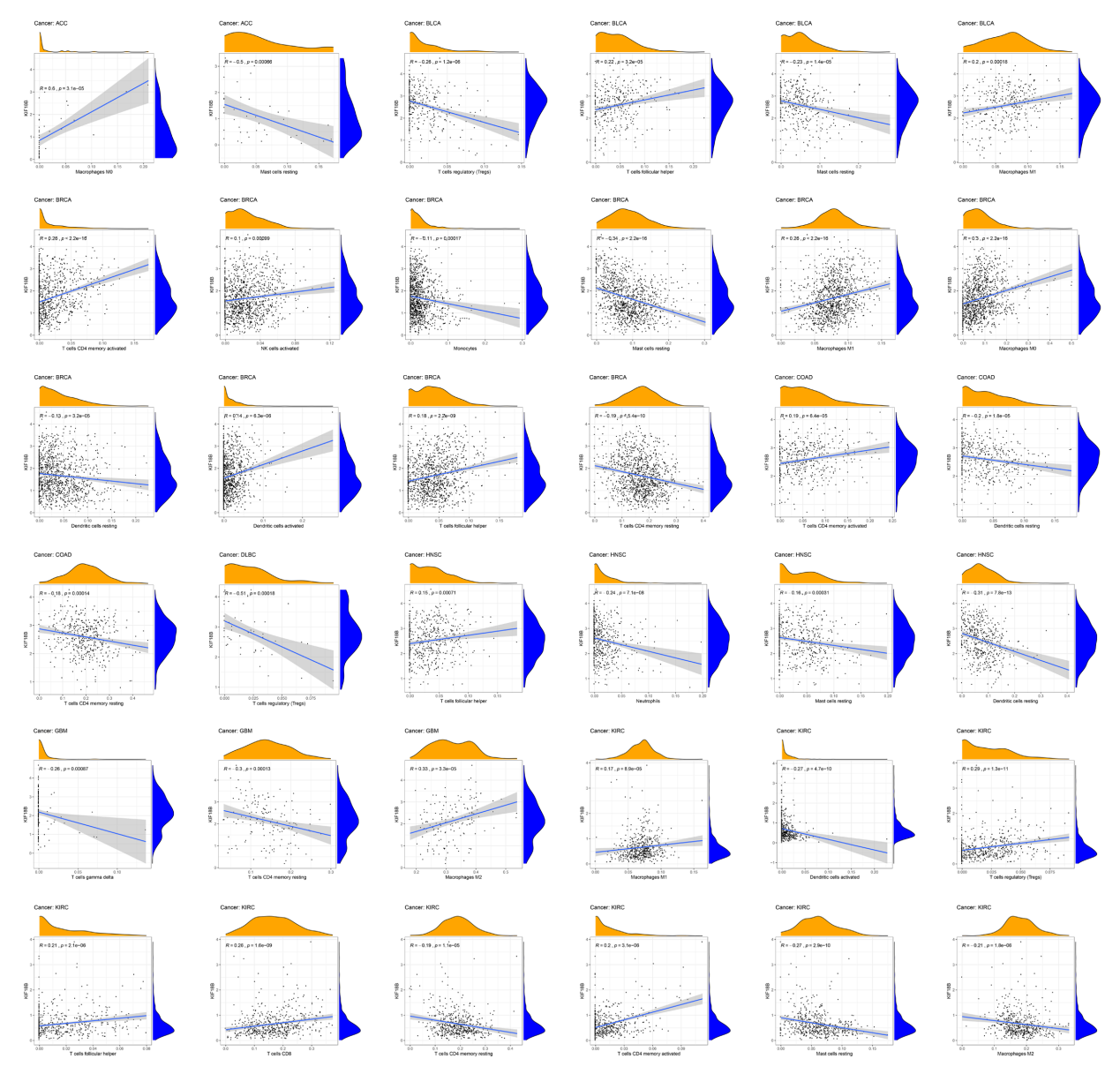


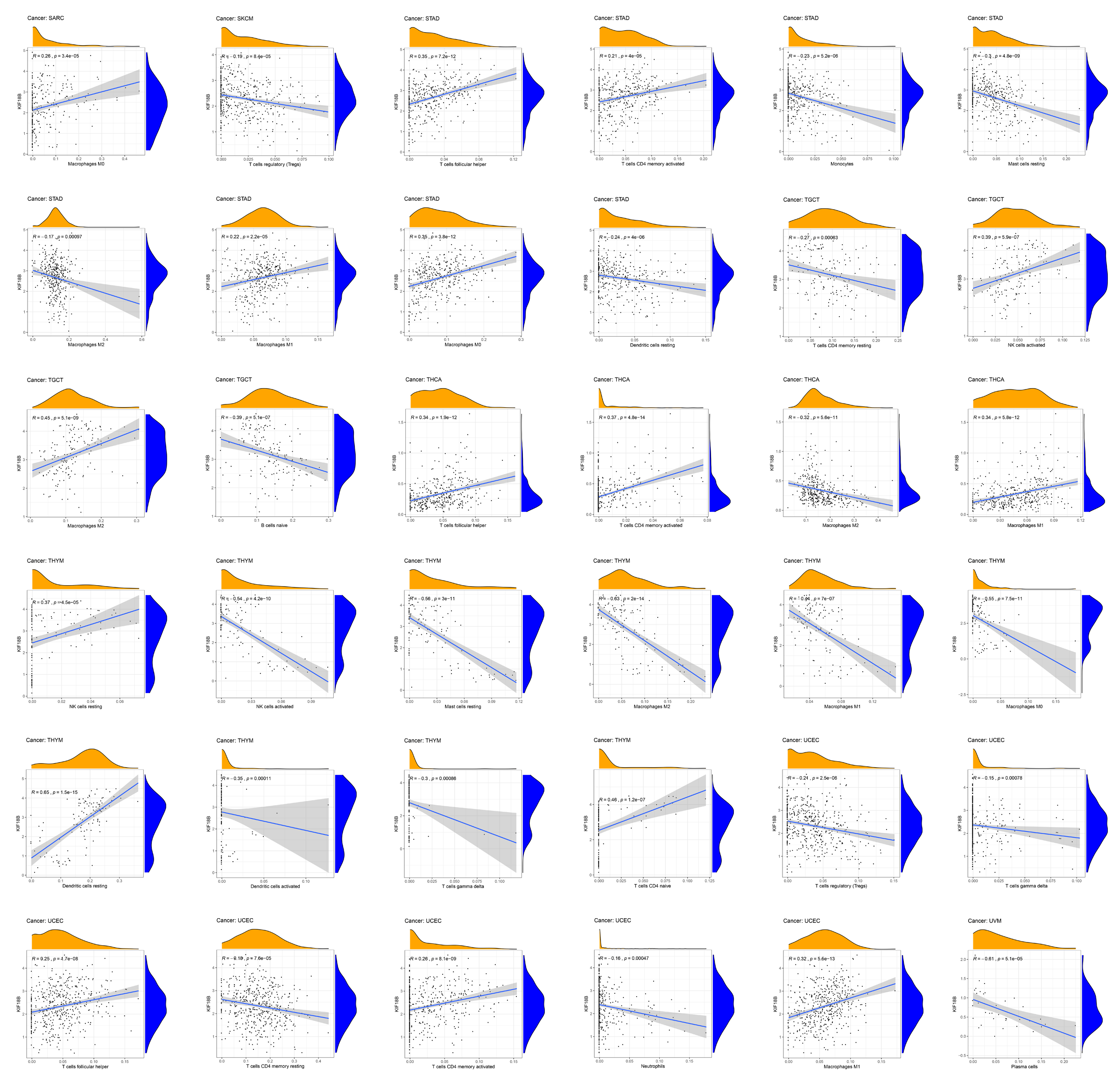


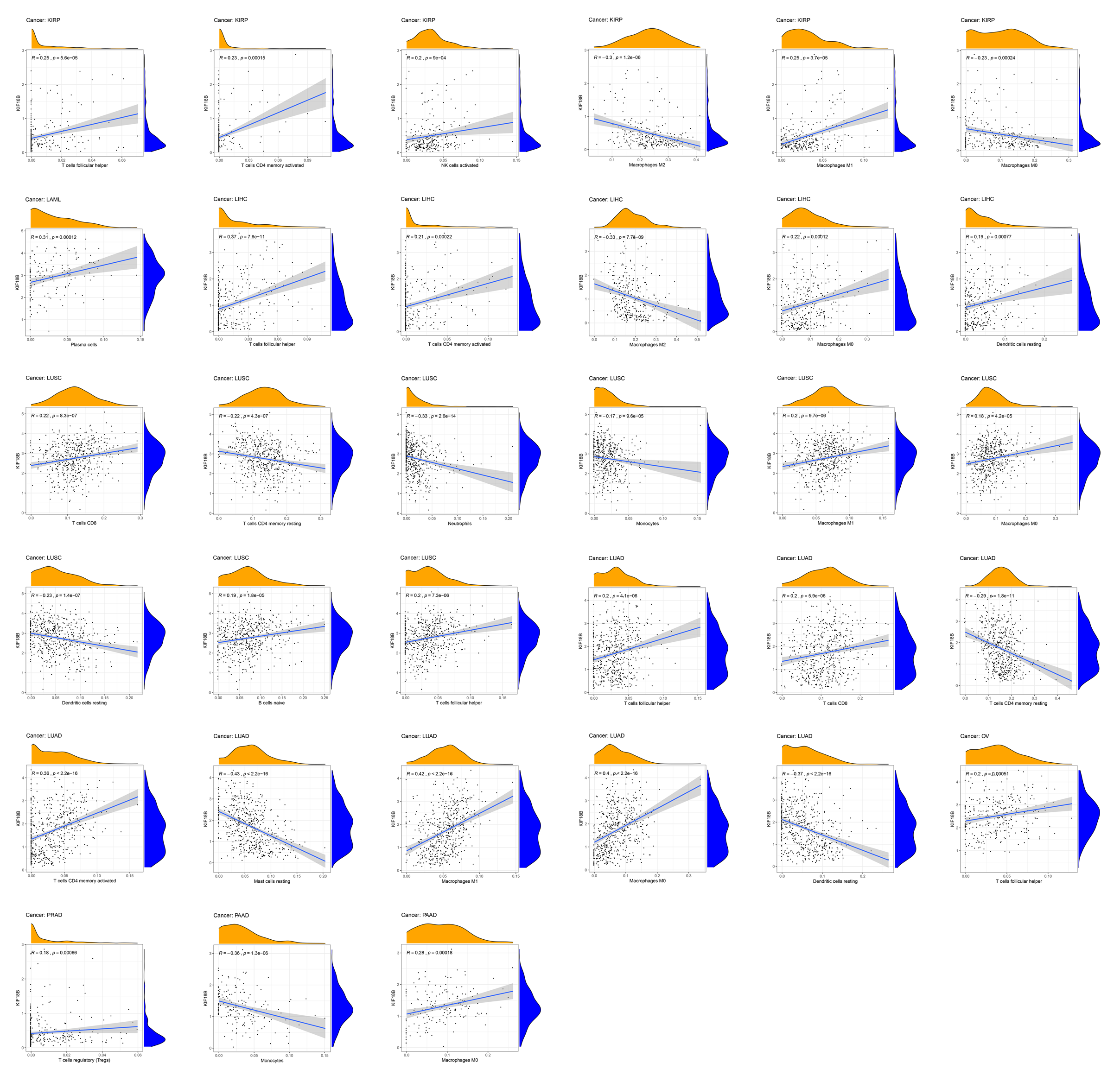


**Supplementary Figure 2.** GO enrichment analysis of KIF18B in various cancers. Values of *P* < 0.05 and results higher than 5 were considered and displayed.


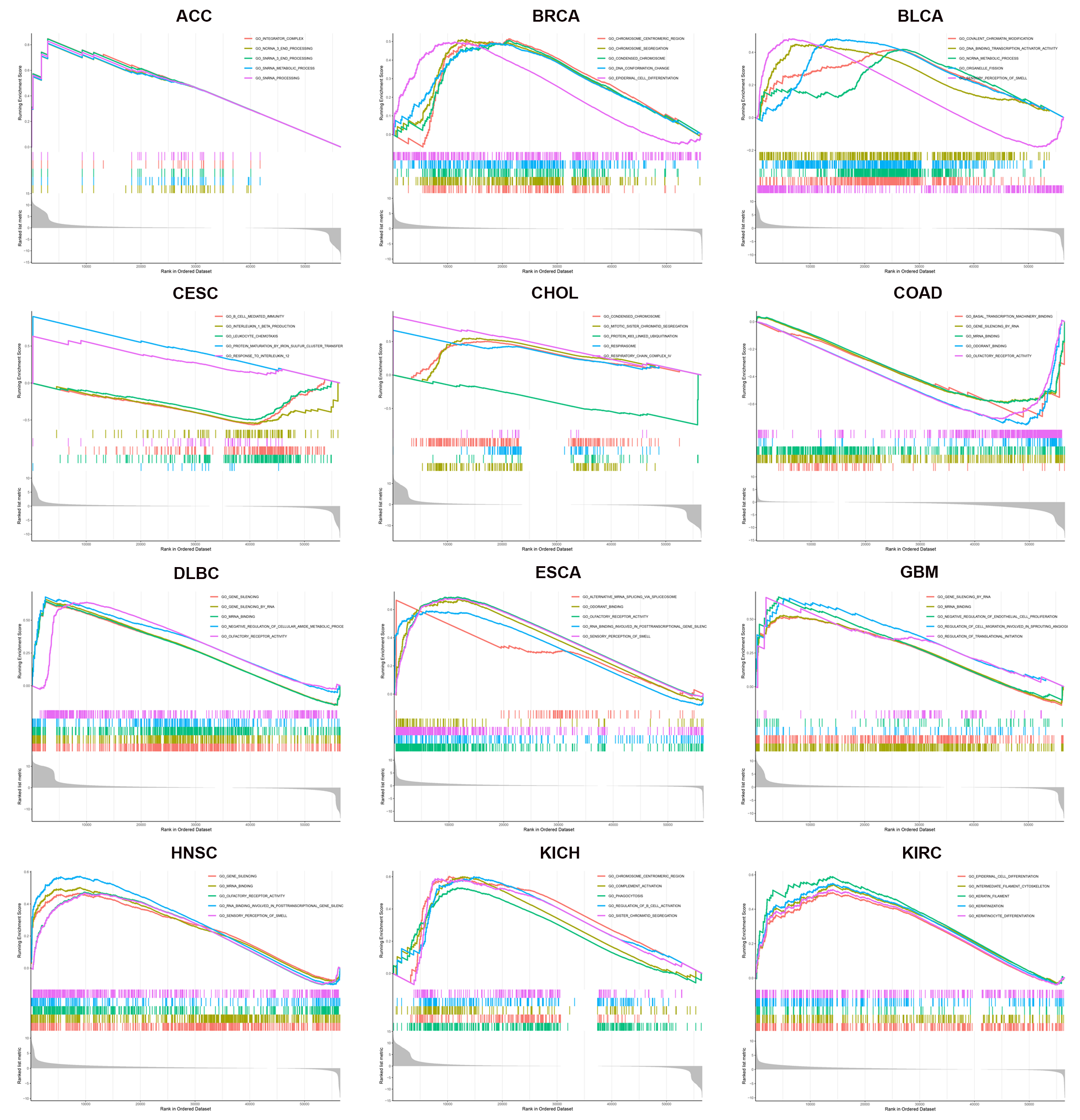


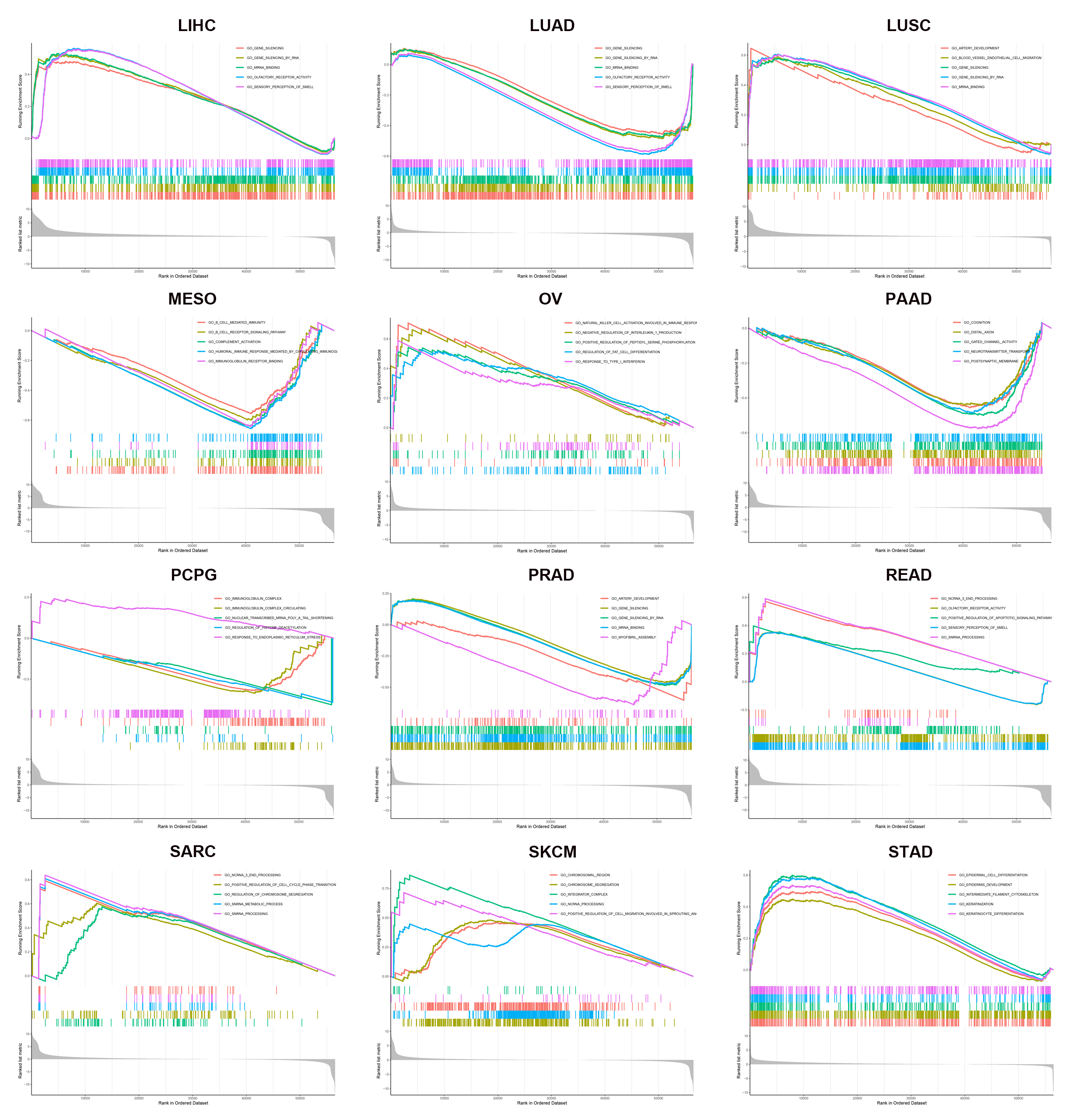


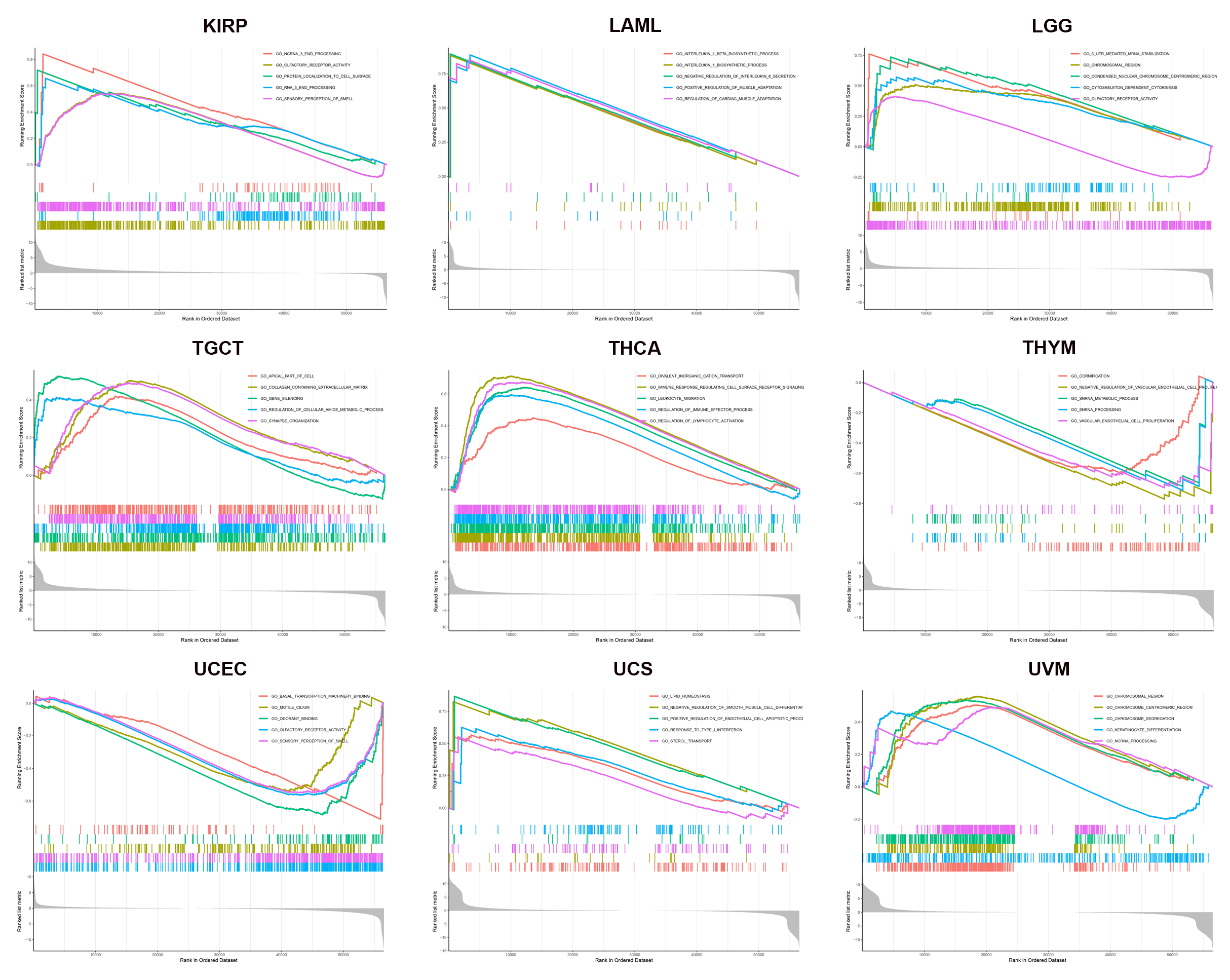

Supplement: Supplementary file 2 [file Table1.DOCX]
